# Strain-induced band modulation and excellent stability, transport and optical properties of penta-MP_2_ (M = Ni, Pd, and Pt) monolayers†

**DOI:** 10.1039/d0na00503g

**Published:** 2020-08-31

**Authors:** Vipin Kumar, Debesh R. Roy

**Affiliations:** Materials and Biophysics Group, Department of Applied Physics, S. V. National Institute of Technology Surat 395007 India vipinkumar0247@gmail.com drr@phy.svnit.ac.in; Hanse-Wissenschaftskolleg (HWK) Lehmkuhlenbusch 4 27753 Delmenhorst Germany

## Abstract

First principle calculations utilizing density functional theory were carried out to investigate the electronic, transport and optical properties of penta-MP_2_ (M = Ni, Pd and Pt) monolayer compounds under applied uniaxial and biaxial tensile strains. With an optimum magnitude of applied strain, we found band gap transitions in penta-MP_2_ monolayers from zero/narrow to the semiconductor regime, wherein band gaps were noticed to be firmly dependent on the applied uniaxial and biaxial tensile strains. In this study, the PBE approach was used primarily to evaluate electronic properties, from where the identified architectures of penta-MP_2_ with maximum obtained bandgaps under respective optimum strains were assessed through the HSE06 method of calculation for better estimation of band gaps and optical properties. Prior to HSE calculations, we affirmed our assessment for the stability and reliability of the compounds under uniaxial and biaxial strains of up to 15% through phonon spectrum and elastic calculations. A distinct transition was also noted from semiconductor to metal for all compounds after the applied optimum uniaxial and biaxial strains. The optical absorption spectra in all the stretched penta-MP_2_ compounds reached the order of 10^6^ cm^−1^, with significant peaks belonging to the IR and visible regions; this indicates promising applications of these materials in high-performance solar energy and good hot mirror materials. The enhanced *I*–*V* responses under uniaxial and biaxial tensile strains using the non-equilibrium Green's function (NEGF) approach confirm the usefulness of the strained state of the considered penta-MP_2_ monolayers. The results show that tuning electronic properties, *I*–*V* characteristics and optical properties of stretched penta-MP_2_ compounds under tensile strain merits significant future applications in optoelectronic devices and as good hot mirror materials.

## Abstract 2

First principle calculations utilizing density functional theory were carried out to investigate electronic properties, transport and optical properties of penta-MP_2_ (M = Ni, Pd and Pt) monolayer compounds under applied uniaxial and biaxial tensile strains.

## Graphical abstract

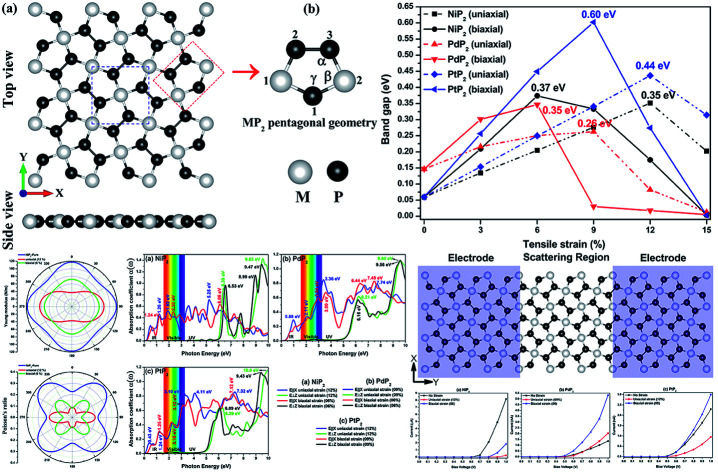

## Introduction

The discovery of graphene in 2004 (ref. 1–3) encouraged substantial scientific efforts in the research community to explore two-dimensional (2D) materials, as they exhibit outstanding physical, mechanical, electronic, optical and transport properties. Various technical developments have contributed to the ongoing explosion in the development of these materials, and various innovative 2D technologies have been introduced.^4–7^ In addition, numerous modern 2D nanostructures have been theoretically proposed with the aid of high-performance computing,^8–11^ and a few of these predicted 2D compounds were successfully fabricated through experiments. Two-dimensional B (boron) and Si (silicene) are among the best examples in this regard.^12–14^ Due to the zero band-gap of graphene, much attention has been paid to investigating 2D semiconductor materials such as phosphorene^15,16^ and transition-metal dichalcogenides.^17,18^ In the recent past, 2D materials with pentagon shapes have also been reported, with fascinating properties. In 2015, penta-graphene was reported by Zhang *et al.*;^19^ it is formed by the strong sp^2^/sp^3^ hybridization of carbon atoms along with high mechanical and dynamical stability up to 1000 K, through AIMD simulations.^19^ Numerous efforts have been made by many researchers to invent 2D materials with penta structures since the successful prediction of penta-graphene.^20,21^ Moreover, 2D group V phosphorene has been successfully separated from bulk black phosphorus;^22^ its bandgap was measured to be about ∼2.0 eV, and possesses a high carrier mobility of up to 10^4^ cm^2^ V^1^ s^1^.^8^ However, due to the issue of rapid degradability in black phosphorus, developed 2D phosphorene derivatives, including GeP_3_,^23^ InP_3_,^24^ δ-InP_3_,^25^ SnP_3_,^26,27^ and CaP_3_,^28^ have been paid immense attention due to expectations of high carrier mobility. Experimentally, a pentagon-shaped PdSe_2_ compound with puckered geometry was successfully exfoliated from its bulk crystal with a bandgap of 1.3 eV and good carrier mobility of ∼158 cm^2^ V^1^ s^1^,^29^ whereas GeP_2_ also showed outstanding results in photocatalysis of the splitting of CO_2_ into CO and of water splitting.^30^ In addition to this work, many other studies have been reported on metallic 2D pentagon-based materials such as penta-graphene,^19^ penta-silicene,^31^ penta-SiN^32^ and penta-CN;^21^ however, it is still exceptionally challenging to achieve wide bandgaps in pentagonal 2D semiconductors. However, it is intriguing to encourage investigation of the potential applications of 2D pentagon-based wide bandgap phosphide materials. Hence, more efforts to explore the potential of pentagon-based compounds may be worthwhile for future useful applications. Qian *et al.*^33,34^ and Yuan H. *et al.*^33,34^ introduced new binary penta transition-metal phosphide/arsenide compounds, MX_2_ (M = Ni, Pd and Pt; X = P and As), which have high carrier mobility with strong optical absorption spectra.^33,34^ The bulk phases of MX_2_-type materials were previously investigated in the 1960s, in which NiP_2_ and PdP_2_ crystals were found to possess monoclinic symmetry with the space group *C*2/*c*, whereas PtP_2_, NiAs_2_, PdAs_2_ and PtAs_2_ are known to possess cubic symmetry.^35^ Studies of these compounds at the DFT-PBE level show a significantly lower band gap (∼0–0.1 eV), which essentially restricts researchers from studying them further in applicable scientific fields. In order to overcome this lower bandgap issue, researchers are continuously engaged in the quest to increase the bandgaps of these materials under various external stimuli for possible applications. Bandgap engineering has been explored through applied external electric field, strain, doping, *etc.*; this has been shown to be a feasible way to control the bandgap, as required for commonly useful low-dimensional systems.^36–40^ The practical use of any two-dimensional material for design and manufacturing generally depends on a better understanding of its strength and mechanical behavior.^41,42^ The optimum strength is the maximum stress under which a perfect crystal can survive at zero temperature while analyze the strength and complete stability of its chemical bonds.^43,44^ In addition, strain can be utilized to tune the bandgap of most 2D compounds. Thus, the elastic limit must be determined to understand how structures differ with stress. In the past, work on blue phosphorene, β-arsenene and β-antimonene has shown that these materials have flexible bandgaps and are strongly dependent on the number of layers, modulated by applied directional strain.^45–49^ It can be understood from the above discussion that tensile strain can be an excellent technique to tune the energy bandgaps of 2D materials and to endow them with useful properties in a versatile range of nanoelectronic and optoelectronic applications.

In the present work, we have performed a systematic investigation of the electronic properties of a series of monolayer penta-MP_2_ (M = Ni, Pd and Pt) compounds under tensile strains of up to 15% using density functional theory (DFT).^50^ Initially, we considered the Perdew–Burke–Ernzerhof (PBE)^51,52^ approach for bandgap modulation to find the maximum bandgap value with respect to a particular amount of strain in the uniaxial and biaxial directions. The enhanced bandgap achieved for a particular amount of strain confirmed the possible existence of the compound in reality under the influence of external strains in the uniaxial and biaxial directions through phonon and mechanical stabilities. Because PBE calculations are known to underestimate bandgap values, we further carried out the popular Heyd–Scuseria–Ernzerhof (HSE06)^53,54^ hybrid calculations to better estimate the bandgap values. The optical properties of the considered materials were also explored with HSE06 calculations. In addition, the current–voltage (*I*–*V*) characteristics of the penta-MP_2_ compounds exhibit isotropic transport properties with and without strain. The present theoretical investigation reveals the bandgap modulation of penta-MP_2_ compounds with remarkably enhanced optical absorption spectra (order of 10^6^ cm^−1^) in the infra-red (IR) and visible ranges of the electromagnetic spectrum, which provides a new direction for their applications in the fields of nanoelectronics and optoelectronics.

### Computational details

The ground state structures and electronic properties of penta-MP_2_ (M = Ni, Pd and Pt) were calculated using density functional theory (DFT)^50^ under the generalized gradient approximation (GGA), as implemented in the Vienna *ab initio* Simulation Package (VASP)^55–58^ with the Perdew–Burke–Ernzerhof (PBE)^51,52^ functional as the exchange–correlation interactions.^58,59^ To understand the electron–ion interactions, we used projector-augmented wave pseudopotentials (PAW).^59,60^ All the calculations were carried out with a plane wave energy cutoff of 550 eV, employed with the Monkhorst–Pack 15 × 15 × 1 *Γ*-centre k-mesh as used for Brillouin zone integrations. The minimum optimization criteria were considered until the total energy convergence reached 10^−7^ eV per atom. Both the ionic position and unit cell were optimized by considering the criteria for maximum residual forces on each atom reaching less than 0.001 eV Å^−1^ under the conjugate gradient method. For geometry optimization and accurate energy band calculations, Fermi-level smearing was taken as 0.01 eV. To avoid interactions between two adjacent layers, we preserved a minimum of ∼20 Å of vacuum space. It is well known that PBE-DFT usually underestimates electronic band gaps. To overcome this issue, we also employed a very popular hybrid functional, Heyd–Scuseria–Ernzerhof (HSE06),^53,54^ to obtain more accurate band gap values. The mixing parameter of the HSE06 functional used in the present work was 25% Hartree–Fock (HF) exchange with 75% PBE exchange, which has been reported to achieve the best possible matches to experimental band gap values in the past:^53,54,61,62^1
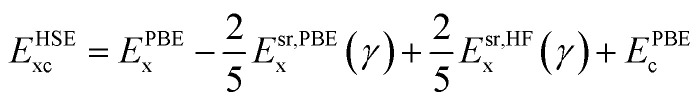


Employing density functional perturbation theory (DFPT)^63^ interfaced with phonopy^64^ code, we evaluated the dynamical strength using the small displacement method of atoms with 4 × 4 × 1 supercells for all the considered structures, and the phonon spectra were determined using the obtained second order force constant. The VESTA^65^ tool was used for visualization and construction of the structures of the materials. In addition, we also investigated the electronic transport properties with the non-equilibrium Green's function (NEGF) methods using Landauer–Buttiker equations as follows:^66,67^2
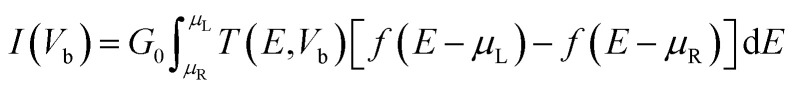
where *G*_0_ is the unit of the quantum conductance, *T*(*E*,*V*_b_) is the transmission probability of incident electrons at a potential bias *V*_b_ and energy *E*, and *μ*_L_ and *μ*_R_ are the electrochemical potentials of the left and right electrodes at a particular voltage bias difference with *eV*_b_ = *μ*_L_ − *μ*_R_. The probability of transmission of electrons from left to right with the given energy *E* was calculated as follows:^68^

3 *T*(*E*,*V*) = *Tr*[*α*_R_(*E*,*V*)*G*^R^(*E*,*V*)*α*_L_(*E*,*V*)*G*^L^(*E*,*V*)*G*^A^(*E*,*V*)]

where *α* and *G*^A^ denote the coupling matrix of the electrodes and the Green's function of the central region, respectively.

## Results and discussion

### Structural properties

**Fig. 1:**
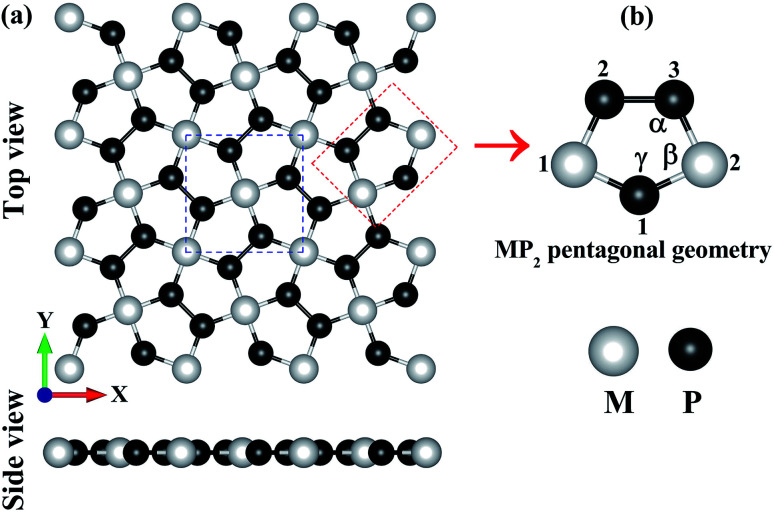
(a) Unit cell of the 2D penta-MP_2_ (M = Ni, Pd and Pt) structure with top and side views, and (b) schematic of the pentagonal geometry of penta-MP_2_.

In Fig. 1, we have shown the structure of the two-dimensional penta-MP_2_ (M = Ni, Pd and Pt) planar monolayer, which has a tetragonal lattice symmetry with pentagonal geometry. In the unit cell as shown in Fig. 1(a) (marked with blue dots), one Ni atom is bonded to four neighboring P atoms, and each P atom is also bonded with another P atom to increase the periodic dimension of the crystal structure. In Table 1, we provide the obtained bond angles and optimized lattice constants for NiP_2_, PdP_2_ and PtP_2_. The lattice constants for NiP_2_, PdP_2_ and PtP_2_ were obtained as 5.57 Å, 5.86 Å and 5.84 Å, respectively, which are consistent with earlier literature reports.^34^ The atoms of penta-MP_2_ were chemically exfoliated from bulk materials, as shown in the blue dotted square box. The red dotted box denotes the pentagonal symmetry of the crystal structure, in which two M atoms are linked with the three P atoms, and all P atoms are in dimers and form P–P bonds. These monolayers can be fabricated from van der Waals crystals as well as from the bulk compounds.^69^ According to Novoselov *et al.*,^1^ mechanical exfoliation is considered to be a suitable technique to obtain graphene monolayer nanosheets from the van der Waals coupled layered bulk graphite structure. A similar chemical exfoliation technique was adopted for chemically bonded bulk phases, such as transition metal nitrides/carbides, known as MXene compounds, to extract their monolayers.^70^ These findings are in line with previous reports, which suggest that the geometry of the MP_2_ monolayer in our present study is reliable. The penta-MP_2_ compounds have been reported to be stable in terms of their dynamical and mechanical aspects.^34^

**Table 1:** The calculated lattice constants (*a*), bond lengths (Å) and bond angles (*θ*) of the penta-MP_2_ (M = Ni, Pd, and Pt) compounds

Compound	*a* (Å)	Bond length (Å)		Angle (*θ*)		
		P_1_–M_1_	P_2_–P_3_	M_1_–P_1_–M_2_ (α)	P_1_–M_2_–P_3_ (β)	M_2_–P_3_–P_2_ (γ)
NiP_2_	5.57	2.18	2.10	129.96	90.00	115.07
PdP_2_	5.86	2.32	2.06	126.65	90.00	116.67
PtP_2_	5.84	2.31	2.08	127.12	90.00	116.44

### Electronic properties

**Fig. 2:**
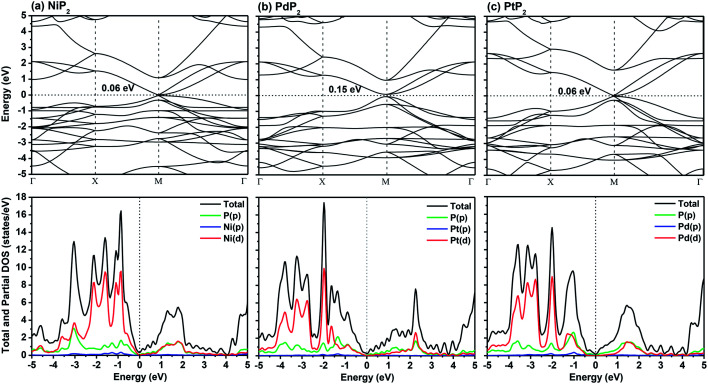
The band structures and partial density of states (PDOS) diagrams of the 2D penta-MP_2_ (M = Ni, Pd, and Pt) compounds (a) NiP_2_, (b) PdP_2_, and (c) PtP_2_.

**Fig. 3:**
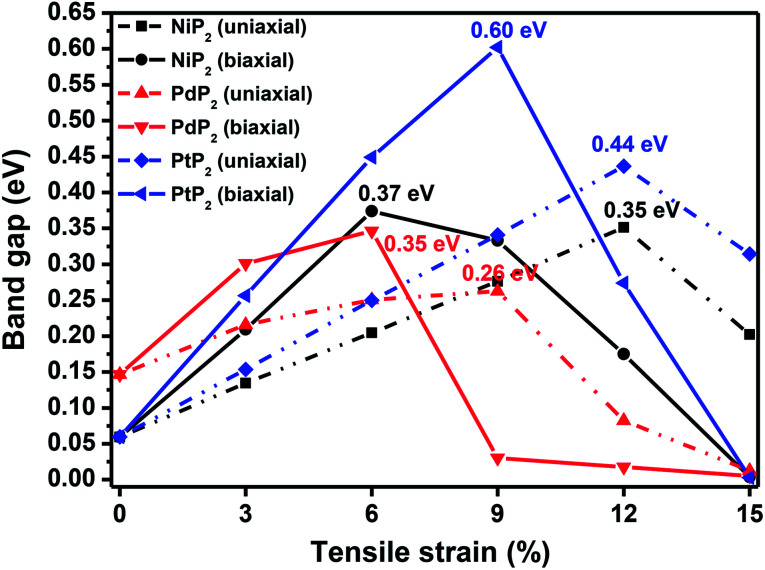
The band gap variations under applied uniaxial and biaxial tensile strains for the 2D penta-MP_2_ (M = Ni, Pd, and Pt) compounds.

In Fig. 2, we present the electronic band structures and partial density of states diagrams of the penta-MP_2_ structures, which reveal very narrow direct bandgaps at the *M*-points of 60 meV, 150 meV and 60 meV for NiP_2_, PdP_2_ and PtP_2_, respectively.^34^ The PDOS analysis shows that the d-orbitals of the transition elements (Ni, Pd and Pt) are making the main contribution near the Fermi level in the valance band maxima (VBM), while the p and d-orbitals show major effects near the Fermi level on the conduction band maxima (CBM). It may be noted here that the narrow bandgaps of penta-MP_2_ create a restriction for their application in the fields of nanoelectronics and optoelectronics. In order to overcome these issues, we applied tensile strains along the uniaxial and biaxial directions of up to 15%. The magnitude of strain was calculated as *S* (%) = [(|*a* − *a*_0_|)/*a*_0_] × 100, where *a*_0_ and *a* are the lattice parameters of the unstrained and strained systems, respectively. Table 2 provides the optimized geometry parameters of the considered penta-MP_2_ compounds for a particular value of the applied uniaxial and biaxial tensile strains at which the bandgap reaches the highest value. The atom–atom bond lengths of M–P and P–P increase from their equivalent positions due to the stretching in the structure, which is responsible for the lower overlap of the electronic orbitals with those of the adjacent atoms. The variation in bandgap values with respect to the applied strains is presented in Fig. 3, where the dotted and solid lines denote the tensile strains along the uniaxial and biaxial directions, respectively.

**Table 2:** Calculated bandgaps (*E*_g_), bond lengths (Å) and bond angles (*θ*) of the 2D penta-MP_2_ (M = Ni, Pd and Pt) compounds at the respective optimum uniaxial and biaxial tensile strains

Compound		NiP_2_		PdP_2_		PtP_2_	
		Uniaxial (12%)	Biaxial (06%)	Uniaxial (09%)	Biaxial (06%)	Uniaxial (12%)	Biaxial (09%)
*E*_g_ (eV)	PBE	0.35	0.37	0.26	0.35	0.44	0.60
	HSE06	0.86	1.30	0.80	0.90	1.02	1.20
Bond lengths (Å)	P_1_–M_1_	2.23	2.32	2.37	2.48	2.36	2.54
	P_1_–M_2_	2.42	—	2.51	—	2.57	—
	P_2_–P_3_	2.12	2.13	2.09	2.10	2.13	2.16
	P_2_–M_1_	2.42	—	2.51	—	2.57	—
	P_3_–M_2_	2.23	—	2.37	—	2.36	—
Bond angles (*θ*)	M_1_–P_1_–M_2_ (α)	127.83	—	125.34	—	125.78	—
	P_1_–M_2_–P_3_ (β)	94.19	90.00	93.30	90.00	93.98	90.00
	M_2_–P_3_–P_2_ (γ)	110.18	116.01	112.09	117.52	111.37	117.47
	P_1_–M_1_–P_2_ (β′)	85.80	—	86.70	—	86.01	—
	M_1_–P_2_–P_3_ (γ′)	121.98	—	122.57	—	112.84	—

**Fig. 4:**
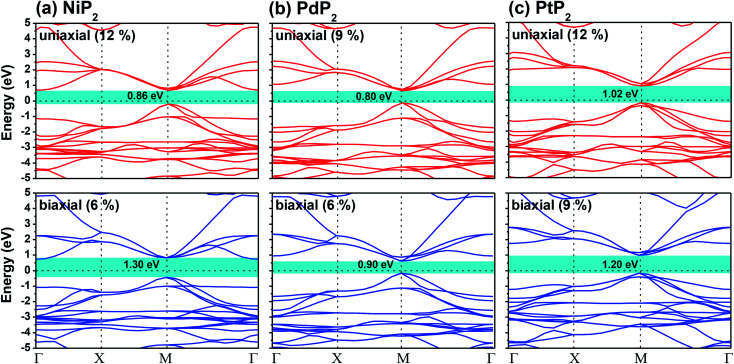
HSE06 functional-calculated band diagrams at the respective optimum uniaxial and biaxial tensile strains for the penta-MP_2_ (M = Ni, Pd, and Pt) compounds (a) NiP_2_, (b) PdP_2_, and (c) PtP_2_.

**Fig. 5:**
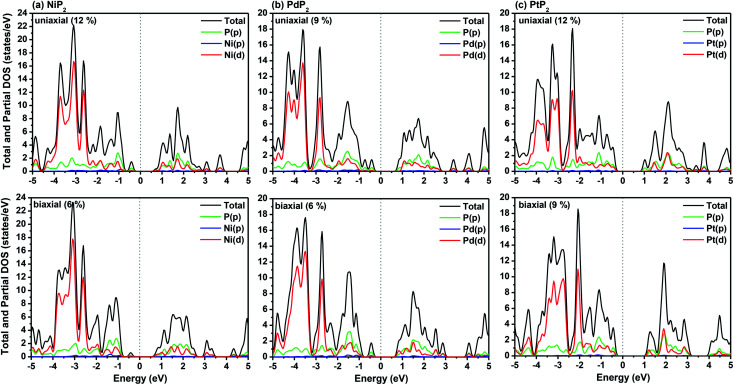
HSE06 functional-calculated partial and total DOS at the respective optimum uniaxial and biaxial tensile strains for the penta-MP_2_ (M = Ni, Pd, and Pt) compounds (a) NiP_2_, (b) PdP_2_, and (c) PtP_2_.

The highest PBE calculated bandgaps for the NiP_2_ compound were found to be 0.35 eV and 0.37 eV after applied tensile strains of 12% (uniaxial) and 6% (biaxial), respectively. For the PdP_2_ case, the PBE bandgap showed the maximum band gap value of 0.26 eV and 0.35 eV at tensile strains of 9% (uniaxial) and 6% (biaxial), respectively. Maximum bandgaps of 0.44 eV and 0.60 eV were observed for PtP_2_ for the applied strains of 12% (uniaxial) and 9% (biaxial), respectively. It was observed that the biaxial tensile strain functions more effectively than the uniaxial strain to reach the maximum bandgap with a lower applied strain effect. Moreover, if the amount of tensile strain increased beyond the critical value of the strain, the bandgap showed a decreasing nature in both cases (biaxial and uniaxial) and achieved the metallic state. The VBM and CBM of the achieved maximum bandgaps were found to be at the same *M*-points in the Brillouin zone, which reveals that all the penta-MP_2_ materials can retain their direct bandgap natures under the applied tensile strains. However, after the transitions of the PdP_2_ and PtP_2_ compounds from semiconductors to semimetals, their VBM and CBM shifted between the *M* and *Γ k*-points. The bandgap variations under applied strains of up to 15% can be understood through the band diagrams of the penta-MP_2_ structures, as shown in Fig. S1–S3 (ESI†). It is currently well known that PBE generally underestimates the values of bandgaps, which do not match experimental results on many occasions. In order to achieve a better estimation of the energy bandgaps, we carried out extensive calculations with the HSE06 functional only for the cases of the maximum bandgaps obtained at particular amounts of tensile strain in the PBE calculations for all the considered penta-MP_2_ compounds. Fig. 4 illustrates the HSE06 band diagrams of the penta-MP_2_ compounds under optimum uniaxial and biaxial tensile strains. The enhanced values of the bandgaps using the HSE calculations were found to be 0.86 eV (1.30 eV), 0.80 eV (0.90 eV) and 1.02 eV (1.20 eV) under uniaxial (biaxial) strains for NiP_2_, PdP_2_ and PtP_2_, respectively, at the *M*-point for all cases. Fig. 5 shows the partial density of states (PDOS) and total density of states (TDOS), wherein the above bandgaps are reflected clearly at the Fermi level. Due to the presence of d-block elements in the penta-MP_2_ compounds, highly intense TDOS peaks were observed. The p-orbitals of the P atoms and d-orbitals of the M (Ni, Pd and Pt) atoms show more contributions in the VBM region compared to the CBM region; meanwhile, up to −2 eV, the P(p) orbital shows a dominant nature in the VBM region near the Fermi level, and the Ni(d) orbital was found to be effective after −2 eV.

In order to check the effects of dipole correction on the electronic properties of our considered systems, we calculated the effects of dipole correction on the total energy (*E*), work function (*ф*) and bandgap (*E*_g_) for all the penta-MP_2_ (M = Ni, Pd and Pt) monolayers under unstrained, uniaxial and biaxial strains, as presented in Table S1 (ESI†). It can be noted from Table S1† that no notable deviations were observed in the total energy (*E*), work function (*ф*), and bandgap (*E*_g_) due to dipole correction for our considered systems.

### Stability of penta-MP_2_ compounds under tensile strains

It is expected that any external physical force will change the properties of a system, which may be effective to endow it with useful properties for different applications. However, it is not necessary for a system to always possess the capacity to tolerate external forces. In the present work, we also examined the application of external force to the equilibrium geometry of the penta-MP_2_ systems, wherein significant energy bandgaps were observed. Therefore, it was also essential for us to investigate the possible stability of the penta-MP_2_ systems under applied tensile strains. Therefore, in order to determine the stability of the penta-MP_2_ monolayers, we obtained the frequency-dependent phonon dispersion curves along the high symmetry paths in the Brillouin region (Fig. S4–S6, ESI†). It is heartening to note that under the tensile strains applied to the penta-MP_2_ structures, no negative frequency was found under the uniaxial and biaxial strains up to of 12% and 9%, respectively, which essentially confirms the stable states for all the maximum bandgaps obtained under the respective uniaxial and biaxial strains. It was noted that in the *Γ*-points, the low-frequency optical and acoustic modes appeared to be well separated from each other. We also observed a continuous decrease of frequency with increasing magnitude of both the uniaxial and biaxial strains, which is also responsible for the lower thermal conductivity. In Fig. S4–S6 (ESI†), the black-marked phonon diagrams illustrate the maximum bandgaps achieved at the optimum applied strains. Overall, the absence of negative frequency under the optimum tensile strains strongly indicates the dynamical stability of our considered penta-MP_2_ (M = Ni, Pd and Pt) compounds.

**Fig. 6:**
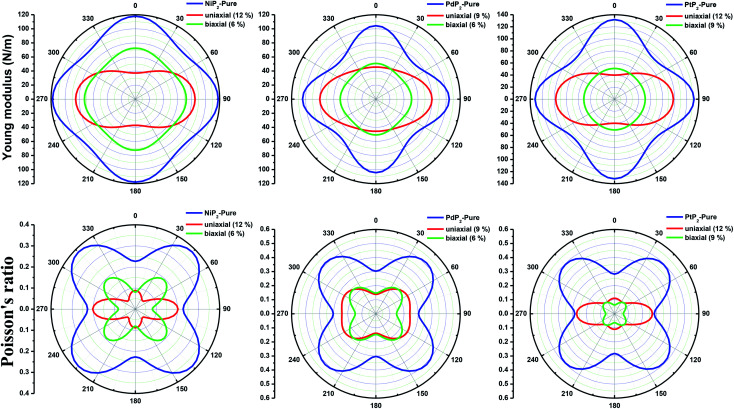
Polar diagrams of the Young's moduli and Poisson's ratios under uniaxial and biaxial tensile strains for the 2D penta-MP_2_ (M = Ni, Pd and Pt) compounds.

Furthermore, we carried out an investigation on the mechanical stability of the considered penta-MP_2_ systems under the optimum tensile strains for which the maximum bandgaps were obtained. The linear elastic constants of the penta-MP_2_ systems were obtained by the finite distortion method.^19^ The Voigt notations (1-*xx*, 2-*yy*, and 6-*xy*)^71^ for biaxial strain-dependent elastic constants have three independent terms of C_11_, C_12_ and C_66_, which denote isotropic nature by having the same symmetry for C_11_ and C_22_, while the uniaxial strain-dependent elastic constants have four independent terms, including C_22_, in which C_11_ ≠ C_22_, which represents anisotropic nature. The in-plane axial Young's moduli and Poisson's ratios for our systems were calculated by the equations *E*_*x*_ = (*C*_11_^2^ − *C*_12_^2^)/*C*_11_; *E*_*y*_ = (*C*_12_^2^ − *C*_12_^2^)/*C*_22_; and *ν*_*x*_ = *C*_12_/*C*_11_; *ν*_*x*_ = *C*_12_/*C*_22_, respectively, and the results are tabulated in Table 3. Because the present materials possess three-dimensional (3D) periodic limits, their two-dimensional elastic constants (*C*_*ij*_) were rescaled by multiplying the *c* factor of the lattice parameter corresponding to the vacuum space between the two conjugative 2D layers,^71–73^*i.e.*, *C*^2D^_*ij*_ = *c* × *C*^3D^_*ij*_. It should be noted that a mechanically stable 2D structure must fulfill the Born criteria, as^72^ (*C*_11_*C*_22_ − *C*_12_^2^) > 0 and *C*_66_ > 0. All the considered penta-MP_2_ compounds were found to be mechanically stable even under applied uniaxial and biaxial strains. The obtained Young's moduli under the strains are remarkably lower than those in the unstrained systems. For NiP_2_, the Young's moduli were found to be 37.78 N m^−1^ (uniaxial) and 72.98 N m^−1^ (biaxial), which are almost 3 and 1.5 times lower than those of the unstrained system. Also, in the cases of PdP_2_ and PtP_2_, the Young's moduli were found to be 2 and 3 times lower in both directions (*x* and *xy*), respectively, compared to their unstrained states. Although the Poisson's ratio (*ν*) slightly differs for all the penta-MP_2_ compounds under uniaxial strain, it is significantly reduced in the case of biaxial strain with respect to the unstrained system. In order to understand and analyze the mechanical properties of the penta-MP_2_, we also obtained polar diagrams of the Young's moduli *E*(*θ*) and Poisson's ratio *ν*(*θ*) using the elastic constant (*C*_*ij*_) as shows in the following equations:^74,75^4
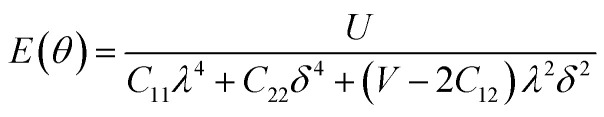
5
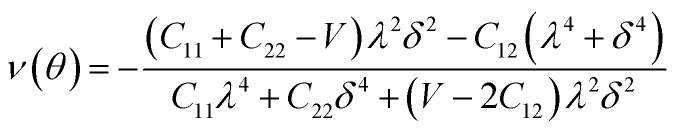
where *λ* and *δ* denote sin(*θ*) and cos(*θ*), respectively, and *U* = *C*_11_*C*_22_ − *C*_12_^2^; *V* = *U*/*C*_66_. As illustrated in Fig. 6, the unstrained and applied-biaxial-strained penta-MP_2_ compounds exhibit isotropic features, while anisotropic features are observed in the case of applied uniaxial strain. It should also be noted from Fig. 6 that the penta-MP_2_ compounds appear to be stiffer under applied tensile strains than the unstrained systems owing to their larger *E*(*θ*). In the case of biaxial strain, all the penta-MP_2_ compounds showed the highest values of *E*(*θ*) for the angles of 0°, 90°, 180° and 360°, similar to the unstrained case; however, because they possess anisotropic features due to the uniaxial strain, the maximum value of *E*(*θ*) lies in the *x*-direction with angles of 90° and 270° while the minimum value lies in the *y*-direction with angles of 0° and 180° for the NiP_2_ and PtP_2_ compounds, respectively. Similarly, the maximum *ν*(*θ*) for penta-MP_2_ is found along the directions of 0°, 90°, 180° and 360° for both no strain and biaxial strain. The maximum *ν*(*θ*) under uniaxial strain lies in the *x* (*y*)-direction of 90° (0°) and 270° (180°) for the NiP_2_ and PtP_2_ compounds, respectively; meanwhile, it was found to be maximum for PdP_2_ along the angles of 60° and 240° in the *x*-direction and minimum along the angles of 0° and 180° in the *y*-direction. Overall, the elastic constants of the penta-MP_2_ compounds under the applied tensile strains indicate their reasonably good elastic properties.

**Table 3:** The calculated elastic constants (*C*_*ij*_), in-plane Young's moduli (*E*) in units of N m^−1^, and Poisson's ratios (*ν*) at the optimum applied uniaxial and biaxial tensile strains respective to the maximum bandgaps

Structure	Strain	C_11_	C_22_	C_12_	C_66_	*E*	*ν*
NiP_2_	Unstrained	124.20	—	28.10	39.30	117.90	0.23
	Uniaxial (12%)	37.78	86.44	7.63	30.27	36.24(*x*), 85.77(*y*)	0.20(*x*), 0.08(*y*)
	Biaxial (06%)	72.98	—	5.87	28.36	72.50	0.08
PdP_2_	Unstrained	114.90	—	34.80	28.40	104.40	0.28
	Uniaxial (09%)	47.18	82.56	11.40	24.89	42.42(*x*), 80.98(*y*)	0.24(*x*), 0.14(*y*)
	Biaxial (06%)	51.93	—	7.65	20.70	50.81	0.15
PtP_2_	Unstrained	142.30	—	40.40	35.90	131.90	0.25
	Uniaxial (12%)	41.12	100.64	11.09	31.90	38.12(*x*), 99.43(*y*)	0.27(*x*), 0.11(*y*)
	Biaxial (09%)	51.12	—	3.21	23.29	50.91	0.06

### Thermal stability of penta-MP_2_ under optimum strains

*Ab initio* molecular dynamics (AIMD) simulations were also carried out in order to investigate the thermal stabilities of our considered penta-MP_2_ (M = Ni, Pd and Pt) compounds under the optimum strains. The change of the total potential energy was monitored during heating processes, and the results show that under the optimum strains of penta-MP_2_, the potential energy fluctuations of these systems are less than 0.5 eV; this confirms the good thermal stabilities of the monolayers at 300 K, as presented in Fig. S7 (ESI†). It is also heartening to note that no bond breaking under the optimum strains of penta-MP_2_ was observed at the end of 5 ps AIMD simulations at 300 K, suggesting significant thermal stability of the penta-MP_2_ compounds under the optimum strains.

### *I*–*V* responses and transmission spectra

**Fig. 7:**
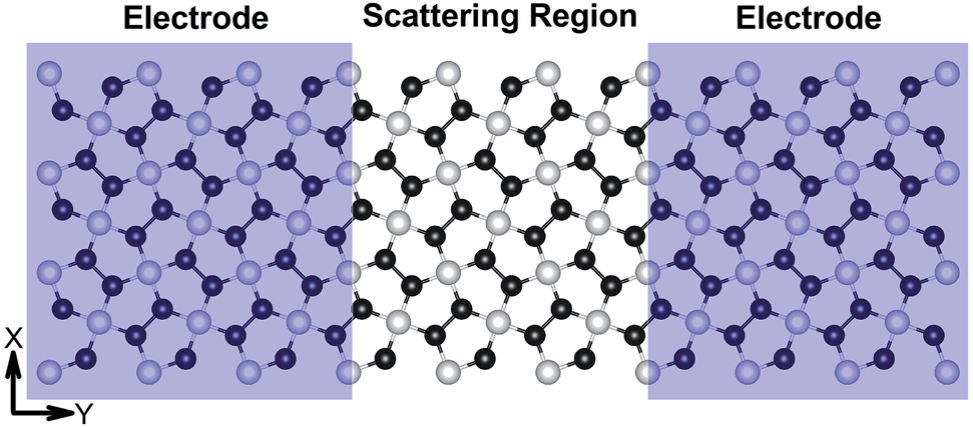
Schematic of the model electronic device setup showing the semi-infinite left and right electrodes (violet-shaded regions) and the central scattering region.

**Fig. 8:**
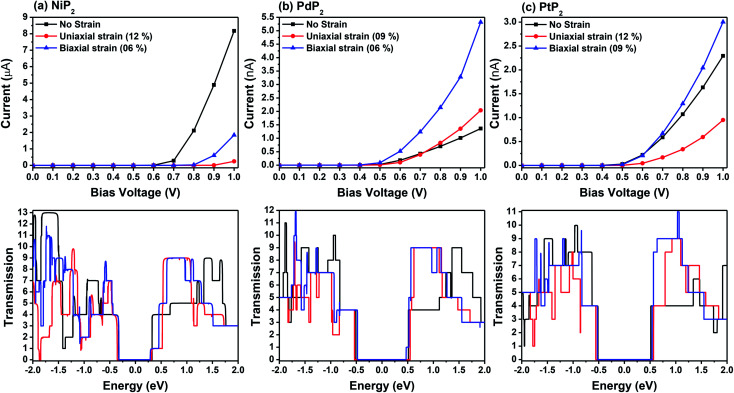
*I*–*V* characteristics and zero-bias transmission spectra for unstrain and under axial tensile strain for the (a) NiP_2_, (b) PdP_2_ and (c) PtP_2_ compounds.

Fig. 7 demonstrates a representation of the two-probe diagram for an electronic transport device for our considered penta-MP_2_ compounds, where two semi-infinite electrodes are coupled with the central scattering region; a 4 × 4 supercell is considered for both the left and right electrodes and for the centre scattering region as well. The 4 × 4 supercell geometry is considered to be the same as that found from the structural optimization and was also used for the electronic property calculations. The transport properties were calculated under the framework of NEGF methods. Fig. 8 illustrates the *I*–*V* characteristics of the penta-MP_2_ systems with and without tensile strain. It was found that for the NiP_2_ compound, although no current response was obtained up to an applied bias of 0.6 V, the response suddenly increased beyond 0.7 V and reached 8.2 μA at 1 V (Fig. 8(a)). Under the applied tensile strain, NiP_2_ do not show any significant impact, especially for the uniaxial strain. For the PdP_2_ and PtP_2_ compounds, as shown in Fig. 8(a and b), although no notable current was found until 0.5 V, remarkable transport was noted for both PdP_2_ and PtP_2_ beyond the applied bias of 0.5 V. In the case of the PdP_2_ compound, 1.5 and 4 times higher current flows (with 2.04 nA and 5.32 nA) at 1 V bias voltage were observed under applied uniaxial and biaxial strains, respectively, whereas for PtP_2_, 4.5 times lower response (0.51 nA) with uniaxial strain and 1.3 times enhancement (3 nA) in current were noted under the biaxial strain at 1 V bias voltage. Overall, it can be concluded that biaxial strain is significantly more effective than uniaxial strain for all the stretched penta-MP_2_ compounds in the present study. The lower vibrational (phonon) frequencies under biaxial strain compared to uniaxial strain may be a reason for these observations, which also accounts for the low thermal conductivity of these compounds. Further, Fig. 8 shows the zero-bias transmission coefficients as a function of energy before and after the applied strains on the penta-MP_2_ systems, where a noticeable effect of the applied tensile strains is observed. Although no significant changes under the tensile strains were noted near the Fermi level, minor transmission peaks for biaxial strain were observed in both the CBM and VBM regions. It should also be noted that biaxial stain shows similar transmission amplitude to the unstrained system, whereas comparatively low amplitude can be noted in the profile of uniaxial strain. The transmission response was found to be considerably high for the penta-MP_2_ compounds under biaxial strain in comparison to uniaxial strain.

### Optical properties of the strained penta-MP_2_ compounds

The optical properties of 2D penta-MP_2_ (M = Ni, Pd and Pt) monolayers under applied uniaxial and biaxial tensile strains are discussed in this section. The dynamical dielectric functions *ε*(*ω*) = *ε*′(*ω*) + *ε*′′(*ω*), including the real *ε*′(*ω*) and imaginary part *ε*′′(*ω*), were determined through HSE06 calculations. *ε*(*ω*) is divided into three parts based on the structural anisotropy, *viz.* the *xx*, *yy* and *zz* directions, with *ε*_*xx*_ = *ε*_*yy*_ ≠ *ε*_*zz*_ and *ε*_*xx*_ > *ε*_*yy*_ ≠ *ε*_*zz*_ for biaxial and uniaxial strains, respectively; therefore, one can understand the different behaviors of the incident light with the polarization of the electric field in-plane (parallel) and out-of-plane (perpendicular). For uniaxial strain, we considered only *ε*_*xx*_ for polarization in-plane (parallel) due to the accumulation of higher relative values than *ε*_*yy*_. The spectra differ mainly due to the fact that *ε*(*ω*) is stronger in the parallel direction in the low energy range in the IR and visible ranges (0 to 3.5 eV), while *ε*(*ω*) intensifies in the perpendicular direction in the high energy UV range (3.5 to 10 eV) based on the blue shifts for the threshold peaks of *ε*(*ω*). Therefore, excitonic effects play an important role in the optical properties and dominate the performance of optoelectronic devices based on two-dimensional materials. The amounts of energy storage and dissipation within the medium are defined by the real *ε*′(*ω*) and imaginary *ε*′′(*ω*) parts, respectively. The *ε*′′(*ω*) is calculated as follows:^76–78^6
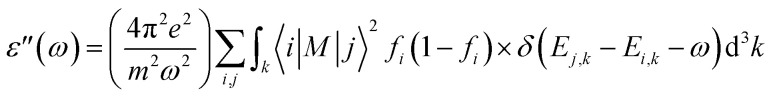
where *ε*, *m*, *ω*, and *M* denote the free electron charge, free electron mass, frequency of the incident photons and dipole matrix, receptively, *i* and *j* represent the initial and final states, respectively, *f*_*i*_ is the Fermi distribution, and *E*_*i*_ is the free electron energy in the *i*^th^ state with wave vector *k*.

The real part of the dielectric function, *ε*′(*ω*), can be obtained using the Kramers–Kronig relationship as follows:^77,79^7
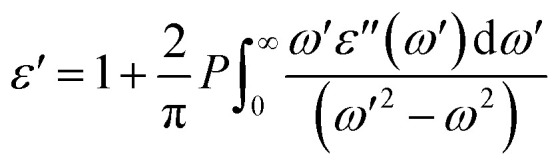
where *P* represents the principal value of the integral. In addition to the dielectric function, we determined the absorption spectra *I*(*ω*), reflectivity *R*(*ω*), energy loss spectra *L*(*ω*) and refractive index *n*(*ω*) using the following equations:^80–82^8
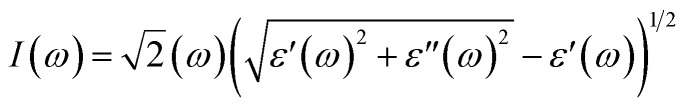
9
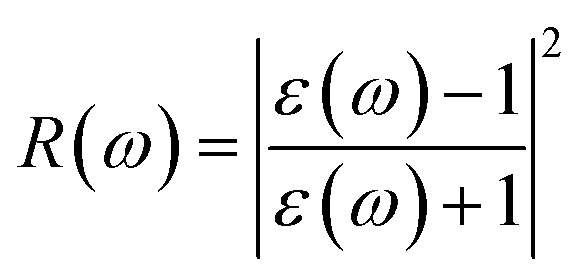
10
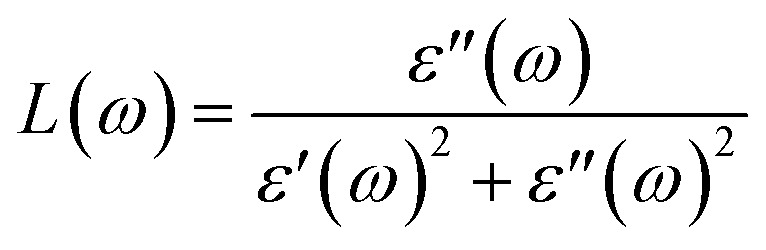
11
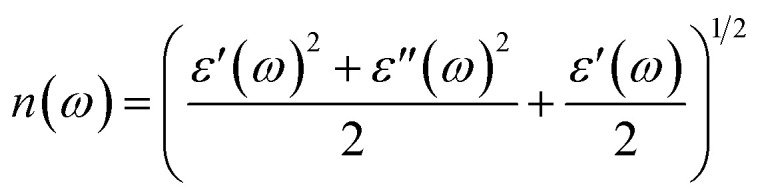


**Fig. 9:**
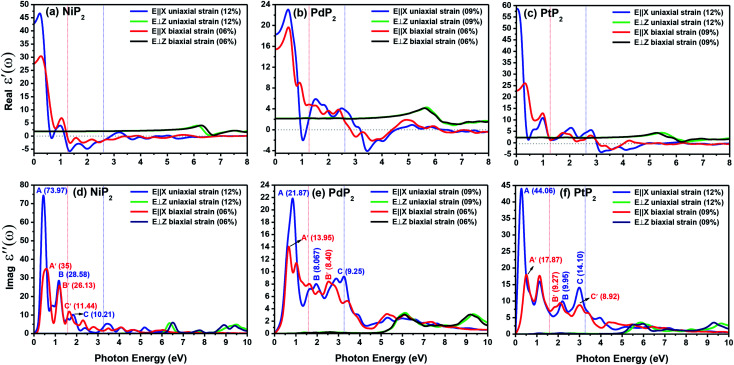
Real and imaginary parts of the dielectric constants of the strained penta-MP_2_ (M = Ni, Pd, and Pt) compounds (a & d) NiP_2_, (b & e) PdP_2_, and (c & f) PtP_2_.

Fig. 9(a–c) present the profiles of the real parts of the dielectric constants of the penta-MP_2_ (M = Ni, Pd, and Pt) compounds under uniaxial (U) and biaxial (B) tensile strains. The calculated static dielectric constants *ε*′(*ω*) in the parallel direction with uniaxial (biaxial) strains are 20.24 (12.61), 18.07 (15.46) and 58.06 (22.64) for NiP_2_, PdP_2_ and PtP_2_, respectively; meanwhile, much lower polarizability was found in the perpendicular direction with the same applied uniaxial and biaxial strains, with values of 1.17, 2.07 and 2.33 for NiP_2_, PdP_2_ and PtP_2_, respectively. Also, the negative values in the dielectric constant of *ε*′(*ω*) indicate metallic nature for a particular amount of incident photon energy in the IR and visible spectra; the same nature was observed in the ultraviolet part of the electromagnetic spectrum. The dielectric constant of *ε*′(*ω*) was found to be negative at 0.68 eV (U), 1.37 eV (U) and 1.39 eV (Bi) for NiP_2_, 1.04 eV (U), 3.44 eV (U) and 3.65 eV (B) for PdP_2_, and 3.17 eV (U), 3.56 eV (B) for PtP_2_ in the parallel direction, while only positive values were observed for the polarization in the perpendicular direction of the electromagnetic (EM) spectrum.

The electron–electron correlation is considered in the imaginary part of the dielectric function *ε*′′(*ω*) for stretched penta-MP_2_ compounds with the use of the HSE functional. In addition, the interactions between electrons and holes (e–h) mostly produce renormalization of the intensity of the optical peak obtained by the HSE functional. The major part of the imaginary dielectric constant *ε*′′(*ω*) shows interband transitions of electrons from the VBM to the CBM, and the bandgap is directly proportional to the energy of the interband transitions near the Fermi energy. It can be noted from Fig. 9(d–f) that the imaginary part of the dielectric constant *ε*′′(*ω*) shows dominant peaks near the band transition energy, as indicated by the peaks A(A′), B(B′) and C(C′) for the uniaxial (and biaxial) tensile strains. The first optical absorption peak was observed in the infrared region (IR) for all compounds, possessing high intensity; this peak showed a redshift in the parallel direction of polarization. This clearly indicates that the stretched penta-MP_2_ compounds are more sensitive in the IR region of the electromagnetic spectrum. In the IR region, the first peak A (uniaxial strain) is much higher than peak A′ (biaxial strain), and biaxial strain shows a blue shift in each case when light is polarized in the parallel direction of the electromagnetic spectrum. This can also be confirmed by the relatively large band gap values observed in the case of biaxial strain compared to uniaxial strain. The peak A(A′) of *ε*′′(*ω*) can be attributed to the interband transition of electrons from the valance band maximum (VBM) to the conduction band minimum (CBM). The second intense peak B(B′) also appears in the IR region for NiP_2_ and PtP_2_, whereas peak B(B′) shows a blue shift in the visible region for the PtP_2_ compound. The third peak C(C′) of *ε*′′(*ω*) is unveiled in the visible region for all the stretched penta-MP_2_ compounds, in which the slightly more intense peak is attributed to the biaxial strain of NiP_2_, and the remaining intense peaks show similar behavior in the PdP_2_ and PtP_2_ compounds. Because the photon energy for visible light ranges from 1.58 to 3.36 eV, this strongly suggests that the stretched penta-MP_2_ monolayers are also strong light-harvesting compounds. All the intense peaks were found to be along the parallel direction in the IR and visible regions for all the stretched penta-MP_2_ compounds, while the peaks along the perpendicular direction were found to be negligible. The light absorption coefficient expresses the percentage of light intensity attenuation per unit distance that is passed in the medium.

**Fig. 10:**
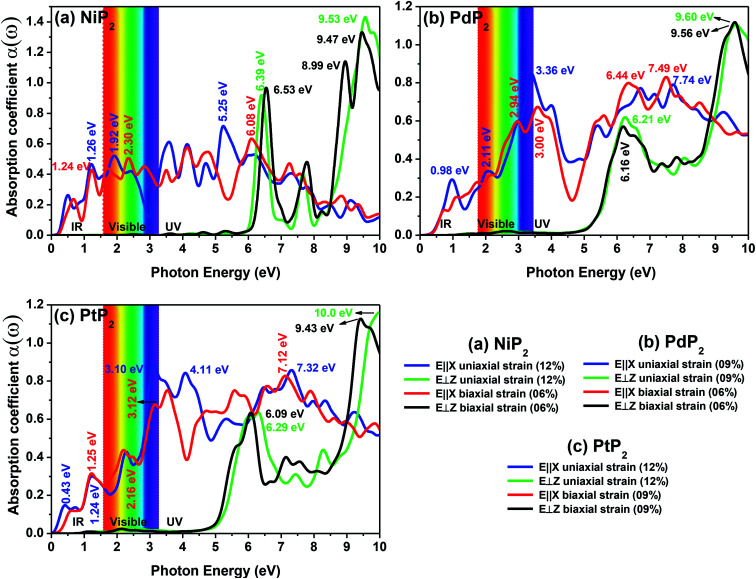
The optical absorption spectra of the stretched penta-MP_2_ (M = Ni, Pd and Pt) compounds (a) NiP_2_, (b) PdP_2_, and (c) PtP_2_.

The optical absorption spectra can be understood by the decay of the incident light intensity spreading in a unit length, as shown in eqn (6). Fig. 10(a–c) shows the obtained absorption spectra for all the stretched penta-MP_2_ compounds to understand the excitation states at various incident energy levels of photons; obvious anisotropy can be observed along different polarization directions. Additionally, the absorption coefficient value reaches an order of 10^6^ for all the cases under uniaxial and biaxial strains, which demonstrates high efficiency in the utilization of solar energy. The planar structure of penta-MP_2_ is mainly due to the interaction between the anti-bonding p_*x*_–p_*y*_ π* orbital of P-dimer and the d_*x*^2^–*y*^2^_ orbital of the transition elements (Ni, Pd and Pt). The absorption spectra were found to be polarized in the parallel and perpendicular directions for the stretched penta-MP_2_ compounds due to the physical effects of the electron–electron (e–e) and electron–hole (e–h) interactions. The optical spectra are entirely re-shaped when the electron–hole interactions are included. The electron–hole interactions show variation when the polarization of light is in a different direction. Hence, the excitonic effect plays a significant role in the optical absorption spectra. A significant amount of optical transition was found at a lower energy range for tensile strain when the light was polarized along the parallel direction, while in the case of the perpendicular direction, optical transitions were found in the higher energy range. The intense absorption peaks are marked in the figure with the respective color of the polarization direction. For NiP_2_, the absorption peaks occur at energies of 1.19 eV (1.21 eV), 1.93 eV (2.23 eV) and 5.21 eV (6.07) in the parallel polarization direction, while for the perpendicular direction, intense peaks are observed only in the high energy region (UV), namely 6.40 eV (6.49 eV) and 9.54 eV (6.49) for uniaxial (biaxial) strain. For PdP_2_, the absorption peaks occur at energies of 0.96 eV, 2.09 eV (2.94 eV) and 3.37 eV (7.49) in the parallel polarization direction, while for the perpendicular direction, relatively high peaks are observed in the UV region of 9.56 eV (9.50 eV) for uniaxial (biaxial) strains. For the case of PtP_2_, the absorption peak arises at energies of 1.22 eV (1.21 eV), 2.30 eV (2.18 eV) and 3.10 eV (3.14) in the parallel polarization direction, whereas for the perpendicular direction, intense peaks are observed only in the higher energy region (UV) of 6.29 eV (9.97 eV) for uniaxial (biaxial) strain. It also can be observed that when the uniaxial strain for polarization was applied in the parallel or perpendicular directions, the optical absorption spectra were red shifted (lower energy range), while for the biaxial strain, blue shifts (higher energy) occurred in the optical absorption spectra. The optical absorption spectra reveal the appearance of stronger peaks in the IR and visible regions under the applied tensile strain compared to the unstrained penta-MP_2_ and other literature-reported penta compounds, *e.g.* PdAs_2_ and PdP_2_.^33,34^

**Fig. 11:**
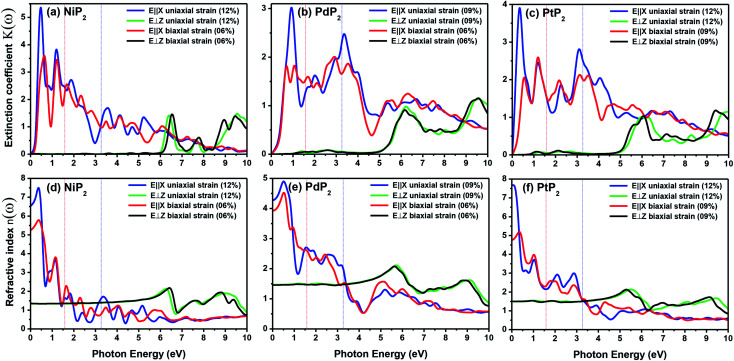
Extinction coefficients and refractive indices of the stretched penta-MP_2_ (M = Ni, Pd and Pt) compounds (a & d) NiP_2_, (b & e) PdP_2_, and (c & f) PtP_2_.

The extinction coefficients and the refraction indices of the stretched penta-MP_2_ compounds are illustrated in Fig. 11. It can be noted from the Fig. 11(a–c) that the first local extinction coefficient *K*(*ω*) maxima in the parallel direction are positioned at 0.47 eV (0.59 eV), 0.85 eV (0.72 eV) and 0.33 eV (1.21 eV) for uniaxial (biaxial) strains, corresponding to the NiP_2_, PdP_2_ and PtP_2_ compounds, and more intense peaks were observed in the IR region for uniaxial strain compared to biaxial strain. The second intense peaks of the extinction coefficient are located at 1.21 eV (1.21 eV), 3.30 eV (2.90 eV) and 1.19 eV (1.19 eV) for uniaxial (biaxial) strains corresponding to NiP_2_, PdP_2_ and PtP_2_. These intense peaks reveal the maximum absorptions in the medium. The photons are absorbed very rapidly in these energy regions. The respective intense peaks in the extinction coefficients have maximum absorption spectra, as presented in Fig. 10(a–c). The static refractive index at 0 eV was found to have the same value of ∼1.5 for all the stretched compounds with polarization in the perpendicular direction, while distinct differences can be observed for the polarization in the parallel direction under uniaxial and biaxial strains. In the parallel direction, the static refractive index under biaxial strain shows lower values than under uniaxial strain, namely 6.50 eV (5.22 eV), 4.26 eV (3.92 eV) and 7.70 eV (4.77 eV) for NiP_2_, PdP_2_ and PtP_2_, respectively. The refractive index *n*(*ω*) in the IR region appears to decrease rapidly with the energy in the parallel direction, whereas it remained steady in the UV region after 7.0 eV, as shown in Fig. 11(d–f). The refractive index was found to be less than 1 over a number of frequencies in the electromagnetic spectrum; this indicates a lower value of light celerity *c* than the light phase velocity, in dissimilarity with the relativity. The signal propagates with the group velocity *v*_g_ = d*ω*/d*k* rather than with the phase velocity (*v*) in a dispersive medium. The relationship between *v*_g_ and *v* is 
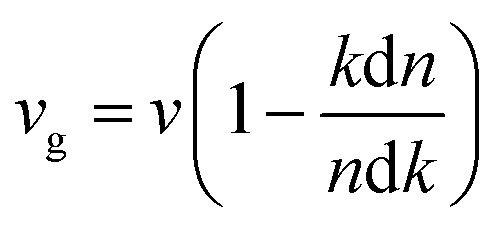
, which indicates that *v*_g_ is always a lower value than *v*.^81^ In the perpendicular direction, it was found to increase slightly monotonically near the UV region, and after fluctuations, it decreased in the far UV region and became constant after ∼15 eV.

**Fig. 12:**
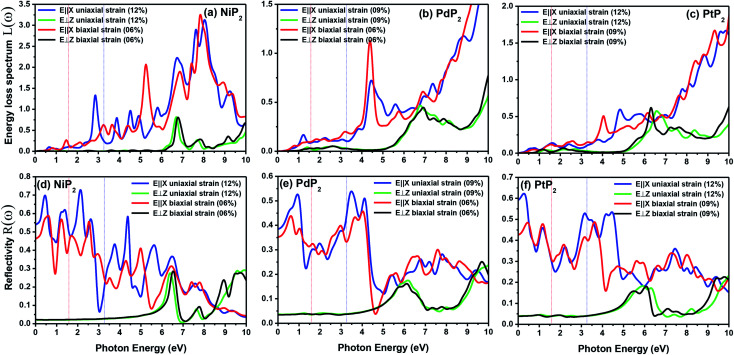
Electron energy loss spectra (EELS) and reflectivities of the stretched penta-MP_2_ (M = Ni, Pd, and Pt) compounds (a & d) NiP_2_, (b & e) PdP_2_, and (c & f) PtP_2_.

The electron energy loss spectrum (EELS) refers to the amount of energy that electrons lose when passing through a dielectric medium, and this can be used to deduce the dielectric functions of materials. The EELS is illustrated in Fig. 12(a–c) as a function of the photon energy for the stretched penta-MP_2_ compounds for the polarization along the parallel and perpendicular directions. The calculated energy loss spectrum *L*(*ω*) for NiP_2_ was found to be quite large at 2.9 eV photon energy (uniaxial direction), whereas a much lower peak was observed at 1.4 eV (biaxial strain) for the polarization in the parallel direction. There are several peaks of EELS, which suggests that the photon loses energy above 4 eV, and resonance is visible in the ultraviolet region. Biaxial strain showed higher EELS peaks compared to uniaxial strain for the polarization along the parallel and perpendicular directions for all the penta-MP_2_ compounds. Intense peaks were observed at 5.34 eV and 7.81 eV for NiP_2_, 4.34 eV and 9.17 eV for PdP_2_, and 4.08 eV and 9.35 eV for PtP_2_ in the parallel direction, while the maxima in the perpendicular direction were found to be 6.81 eV, 6.67 eV and 6.30 eV for NiP_2_, PdP_2_ and PtP_2_, respectively. The lower effect of the EELS below 3.5 eV strongly suggests that our compounds are more subtle in the IR and visible regions.

The reflectivities *R*(*ω*) of the stretched penta-MP_2_ compounds in both directions of polarization are demonstrated in Fig. 12(d–f), which specifies how many photons are reflected from the material and incident on the material. For all photon energies, the reflection was found to be much higher in the parallel direction due to stronger optical excitation than in the vertical direction for all compounds. In comparison to the PdP_2_ and PtP_2_ compounds, the maximum reflectivities were observed in the NiP_2_ compound to be 70% (57%), 73% (54%) and 58% (40%) for uniaxial (biaxial) strains in the electromagnetic spectra. Taking into account the above observations, NiP_2_ appears to be a much better IR and visible light absorber, while PdP_2_ and PtP_2_ are appropriate as IR and UV light absorbers. Overall, due to the higher reflectivity and considerable absorption in the IR and visible regions, NiP_2_ appears to be a non-transparent material. Moreover, the quite low adsorption peaks (Fig. 10) and high reflectivity peaks (Fig. 12(d–f)) in the IR region of the spectrum for all the stretched penta-MP_2_ compounds suggest that these compounds will not absorb much heat, and they can be used as good hot mirrors for application in heat control, environmental protection, coatings, *etc.*^83–85^ For the sake of completeness, we also calculated the optical properties of the penta-MP_2_ (M = Ni, Pd and Pt) monolayers without strain, as presented in Fig. S8 (ESI†), for comparison. In the case of the stretched penta-MP_2_ compounds, we observed some additional adsorption peaks in the IR region, whereas the intensity of the peaks increased in the visible region after the application of uniaxial strain in comparison to the unstrained penta-MP_2_ case.

## Concluding remarks

In summary, the structural, electronic, *I*–*V* and optical properties of the stretched penta-MP_2_ (M = Ni, Pd and Pt) compounds were systematically investigated in detail using first-principles calculations. The present investigation reveals that the application of uniaxial and biaxial strains enhances the energy band gap significantly for the considered penta-MP_2_ compounds, which possess nearly zero band gaps in their original state. Our results show that under optimum amounts of applied uniaxial and biaxial strain, the band gap appears to be maximal, after which the materials proceed toward metallic nature. The stability of the considered penta-MP_2_ compounds under strain was confirmed through phonon dispersion and elastic coefficient analyses. The implementation of HSE06 calculations for determining the electronic and optical properties provides more reliability in the obtained results. The *I*–*V* characteristics of the stretched penta-MP_2_ compounds show better transport characteristics than the unstretched systems for the PdP_2_ and PtP_2_ systems. The electronic and optical properties of penta-MP_2_ were found to be modulated effectively by tensile strains through the changes in the p orbitals of the P atom and the d_*x*_–d_*y*_ orbital of the transition element (Ni, Pd or Pt). The optical absorption spectra for all the compounds reached 10^6^ cm^−1^ under the applied strains, and intense peaks were found in the IR and visible region, which indicates the possible usage of these materials for high performance solar energy and good hot mirror material applications. The higher reflectivity and considerable absorption in the IR and visible regions suggest the non-transparent nature of the considered penta-MP_2_ materials. The outcomes of the present investigation not only reflect great potential applications for the stretched penta-MP_2_ compounds as ultra-thin reflectors and good absorbers for optoelectronic applications, but may also direct the discovery of new optical information and realization of various optical functions in specific frequency ranges of the electromagnetic spectrum. The developed penta-MP_2_ monolayers can also be used as good hot mirrors in various applications, such as heat control, environmental protection, and coatings.

## Conflicts of interest

The authors declare that they have no conflict of interest.

## Supplementary Material

NA-002-D0NA00503G-s001
