# Supplementary material for: Strain-induced band modulation and excellent stability, transport and optical properties of penta-MP2 (M = Ni, Pd, and Pt) monolayers
Source: Nanoscale Adv. 2020 Aug 31;2(10):4566–80. doi: 10.1039/d0na00503g (PMC9417837; doi:10.1039/d0na00503g)
Supplement: NA-002-D0NA00503G-s001 [file NA-002-D0NA00503G-s001.pdf]

Electronic Supplementary Material (ESI) for Nanoscale Advances.
This journal is © The Royal Society of Chemistry 2020

SUPPLEMENTARY INFORMATION

Strain induced band modulation and excellent stability, transport and optical properties of Penta-MP_2 (M = Ni, Pd and Pt) monolayers

Vipin Kumar[1,*] and Debesh R Roy[1,2,*]

[1]*Materials and Biophysics Group, Department of Applied Physics, S. V. National Institute of Technology, Surat 395007, India*
[2]*Hanse-Wissenschaftskolleg (HWK), Lehmkuhlenbusch 4, 27753 Delmenhorst, Germany*

*Email: vipinkumar0247@gmail.com (VK); drr@phy.svnit.ac.in (DRR)

CONTENTS

Table S1: The calculated total energy (E), work function (ϕ) and PBE bandgap (E_g) with and without consideration of the dipole correction. [P. **S2**]

Fig. S1: Band structures of the Penta-NiP_2 monolayer under different uniaxial and biaxial strains. The first and second rows represent the uniaxial and biaxial strains, respectively, whereas black color band diagrams indicate the band structure respective to the maximum energy gap under uniaxial and biaxial strains. [P. **S3**]

Fig. S2: Band structures of the Penta-PdP_2 monolayer under different uniaxial and biaxial strains. The first and second rows represent the uniaxial and biaxial strains, respectively, whereas black color band diagrams indicate the band structure respective to the maximum energy gap under uniaxial and biaxial strains. [P. **S4**]

Fig. S3: Band structures of the Penta-PtP_2 monolayer under different uniaxial and biaxial strains. The first and second rows represent the uniaxial and biaxial strains, respectively, whereas black color band diagrams indicate the band structure respective to the maximum energy gap under uniaxial and biaxial strains. [P. **S5**]

Fig. S4: Phonon diagram of the Penta-NiP_2 monolayer under different uniaxial and biaxial strains. The first and second rows represent the uniaxial and biaxial strains, respectively. [P. **S6**]

Fig. S5: Phonon diagram of the Penta-PdP_2 monolayer under different uniaxial and biaxial strains. The first and second rows represent the uniaxial and biaxial strains, respectively. [P. **S7**]

Fig. S6: Phonon diagram of the Penta-PtP_2 monolayer under different uniaxial and biaxial strains. The first and second rows represent the uniaxial and biaxial strains, respectively. [P. **S8**]

Fig. S7: The AIMD profile of fluctuation in total potential energy (E_{tot}) and temperature for both the uni- and biaxial optimum strains of the Penta-MP_2 compounds at 300 K temperature. The snapshots represented are at the end of 5 ps AIMD simulations at 300 K. [P. **S9**]

Fig. S8: The profiles of optical properties of Penta-MP2 (M=Ni, Pd, Pt) monolayers without strain: (a) Absorption coefficient (10^5), (b) Energy loss spectrum, (c) Reflectivity and (d) Refractive index. [P. **S10**]

Table S1: The calculated total energy (E), work function (ϕ) and PBE bandgap (E_g) with and without consideration of the dipole correction.

Structure	Strain	E (eV)		ϕ (eV)		E_g (eV)	
		without	with	without	with	without	with
**NiP_2**	Unstrained	-32.79	-32.83	5.30	5.30	0.06	0.06
	Uniaxial (12%)	-31.42	-31.46	5.05	5.22	0.35	0.35
	Biaxial (06%)	-31.82	-31.86	5.08	5.23	0.33	0.35
**PdP_2**	Unstrained	-32.53	-32.55	5.14	5.14	0.15	0.15
	Uniaxial (09%)	-31.75	-31.78	5.09	5.05	0.26	0.26
	Biaxial (06%)	-31.63	-31.65	5.13	5.10	0.35	0.35
**PtP_2**	Unstrained	-35.52	-35.53	5.39	5.40	0.06	0.06
	Uniaxial (12%)	-33.80	-33.82	5.43	5.42	0.44	0.44
	Biaxial (09%)	-33.08	-33.09	5.43	5.45	0.60	0.60

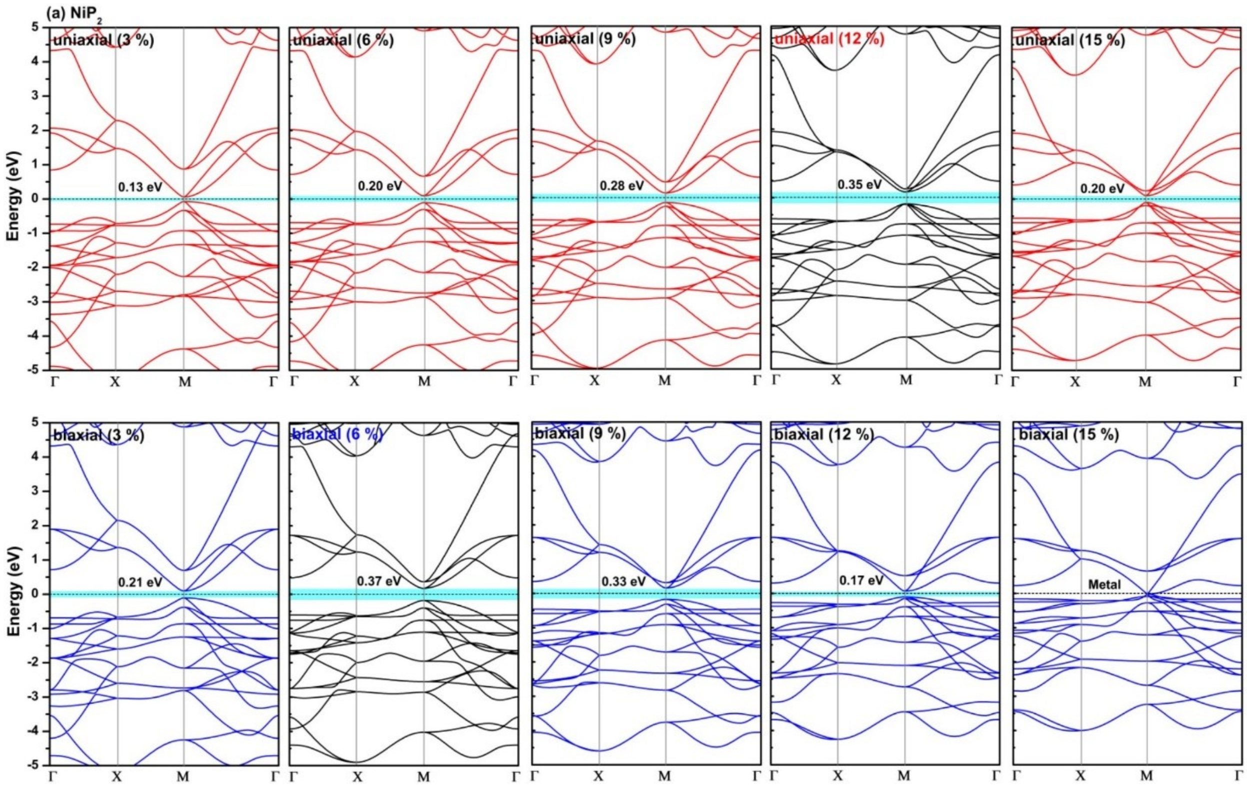

Fig. S1: Band structures of the Penta-NiP_2 monolayer under different uniaxial and biaxial strains. The first and second rows represent the uniaxial and biaxial strains, respectively, whereas black color band diagrams indicate the band structure respective to the maximum energy gap under uniaxial and biaxial strains.

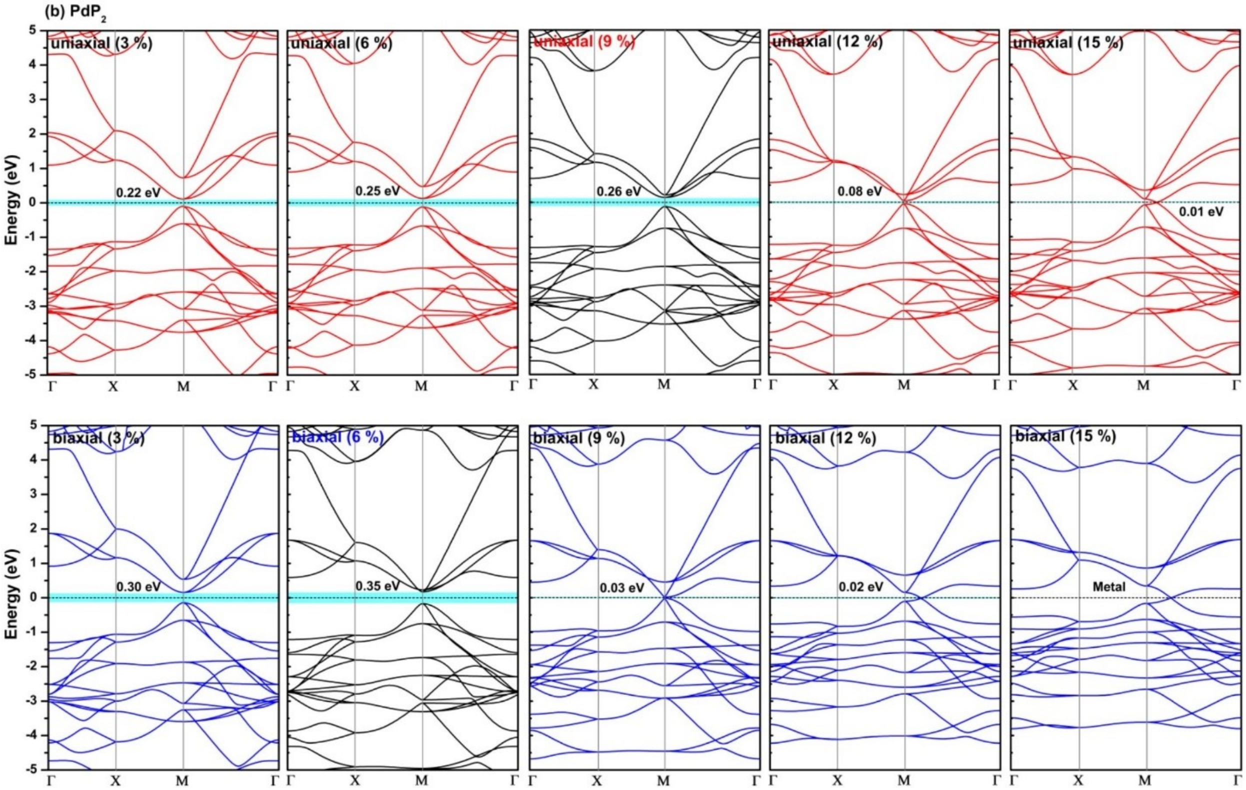

Fig. S2: Band structures of the Penta-PdP_2 monolayer under different uniaxial and biaxial strains. The first and second rows represent the uniaxial and biaxial strains, respectively, whereas black color band diagrams indicate the band structure respective to the maximum energy gap under uniaxial and biaxial strains.

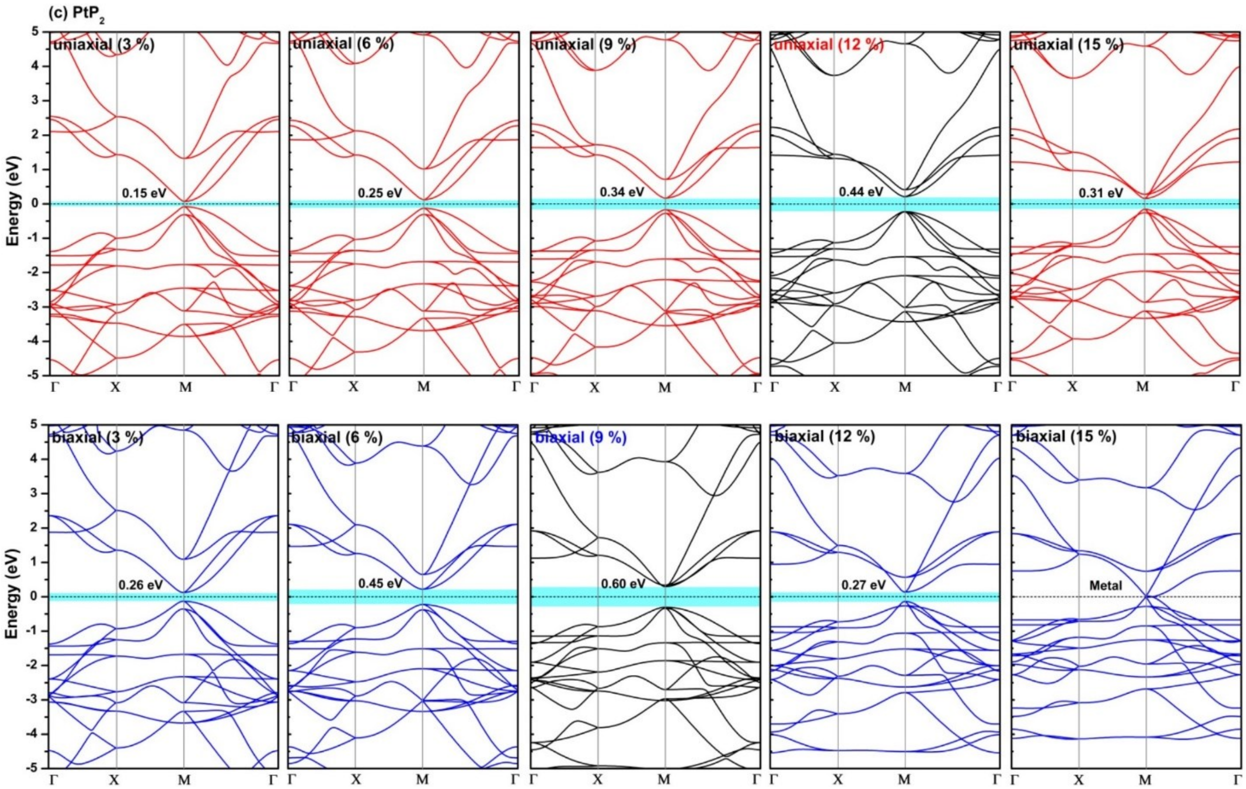

Fig. S3: Band structures of the Penta-PtP_2 monolayer under different uniaxial and biaxial strains. The first and second rows represent the uniaxial and biaxial strains, respectively, whereas black color band diagrams indicate the band structure respective to the maximum energy gap under uniaxial and biaxial strains.

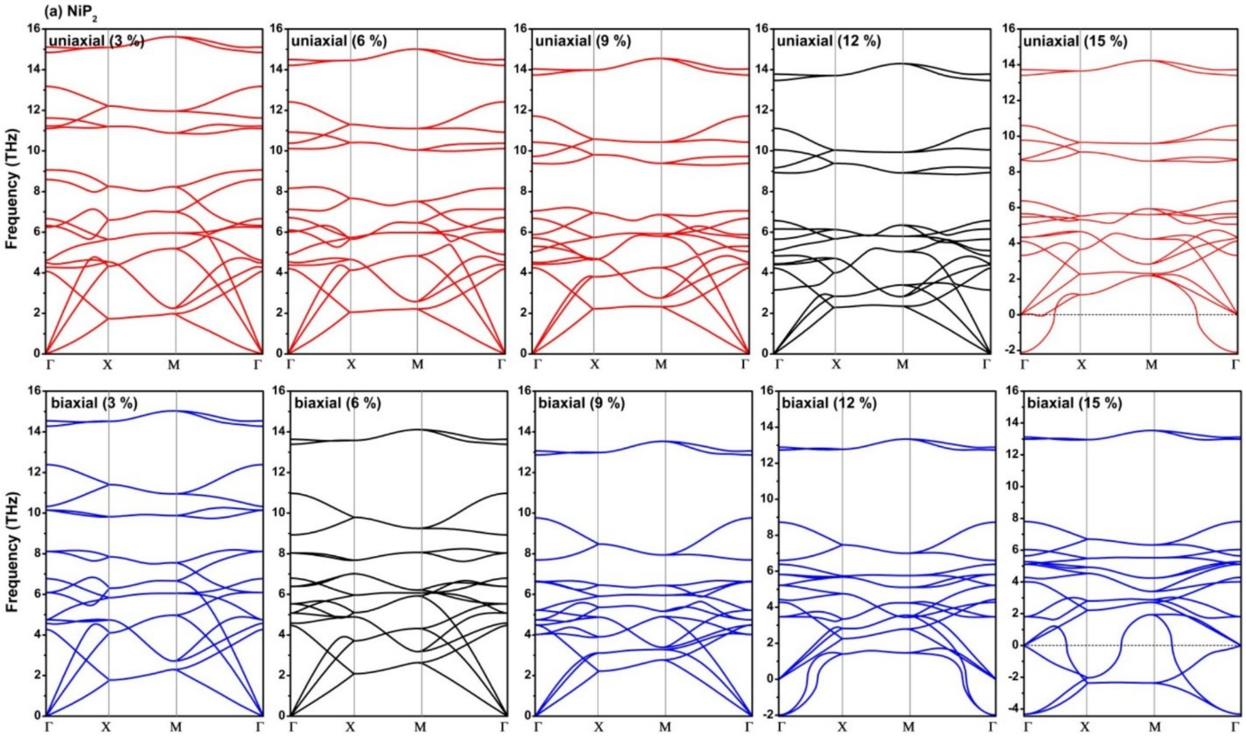

Fig. S4: Phonon diagram of the Penta-NiP_2 monolayer under different uniaxial and biaxial strains. The first and second rows represent the uniaxial and biaxial strains, respectively.

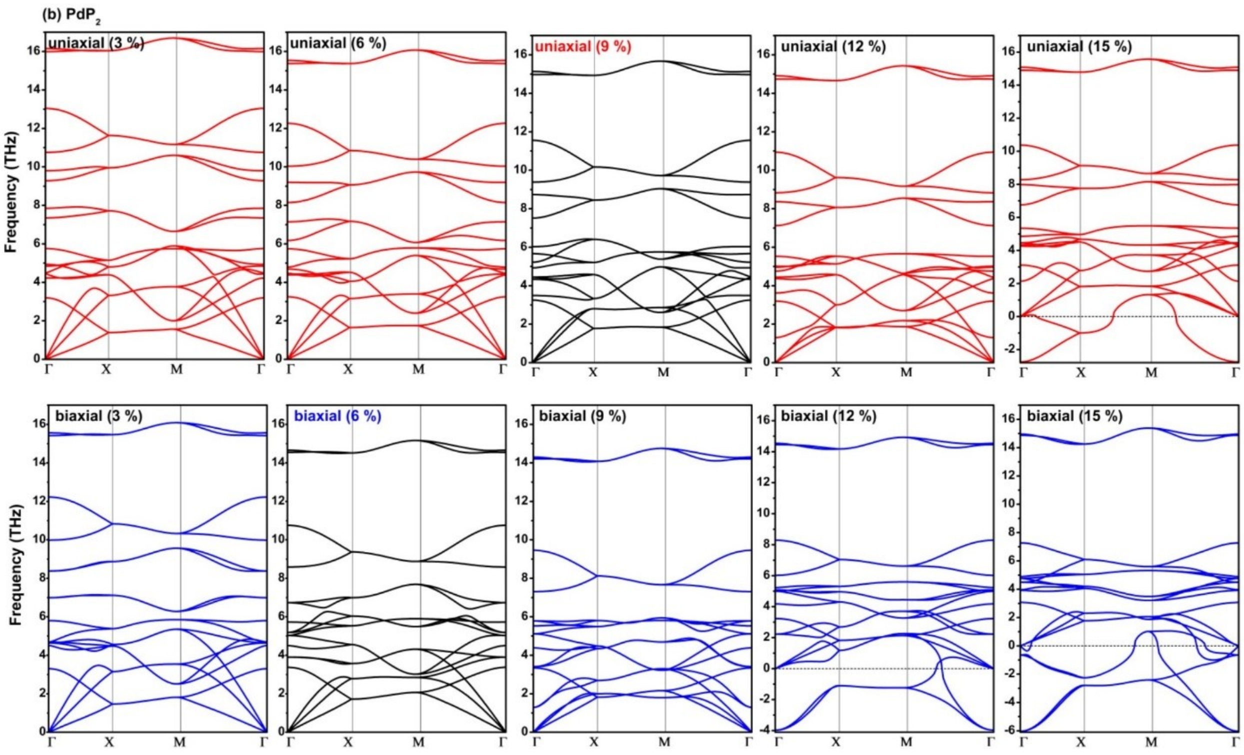

Fig. S5: Phonon diagram of the Penta-PdP_2 monolayer under different uniaxial and biaxial strains. The first and second rows represent the uniaxial and biaxial strains, respectively.

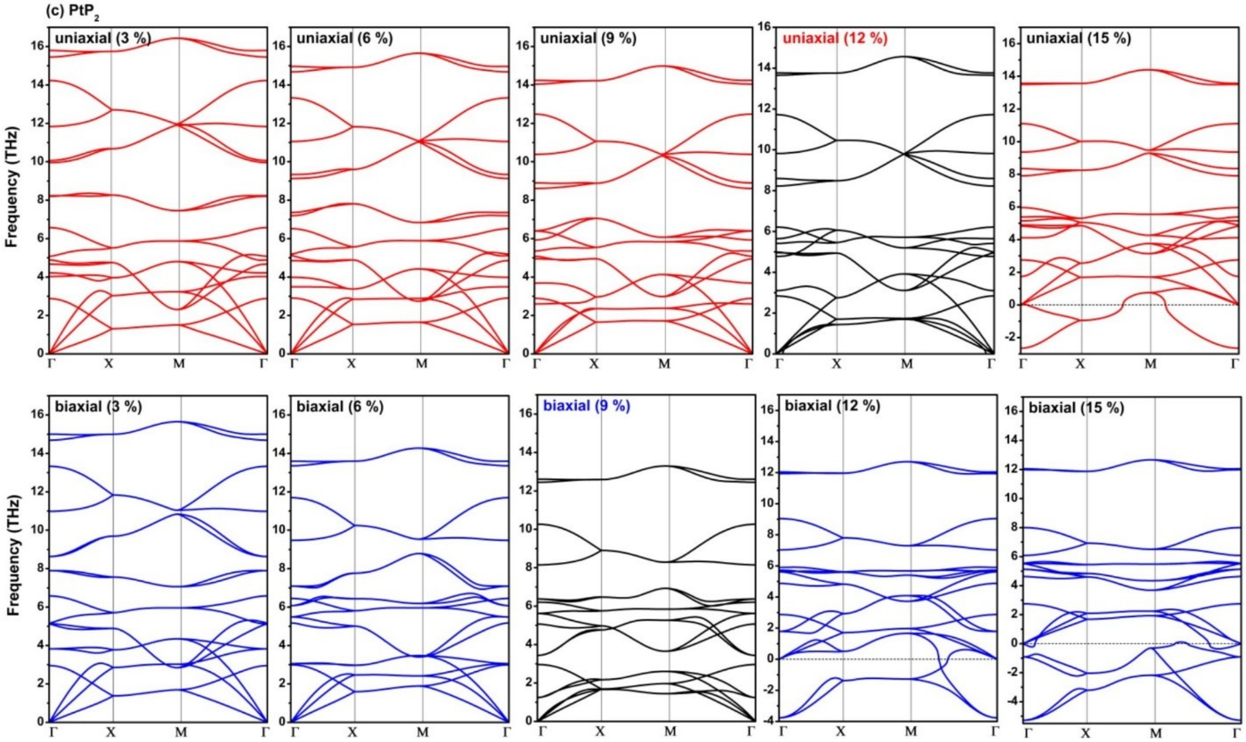

Fig. S6: Phonon diagram of the Penta-PtP_2 monolayer under different uniaxial and biaxial strains. The first and second rows represent the uniaxial and biaxial strains, respectively.

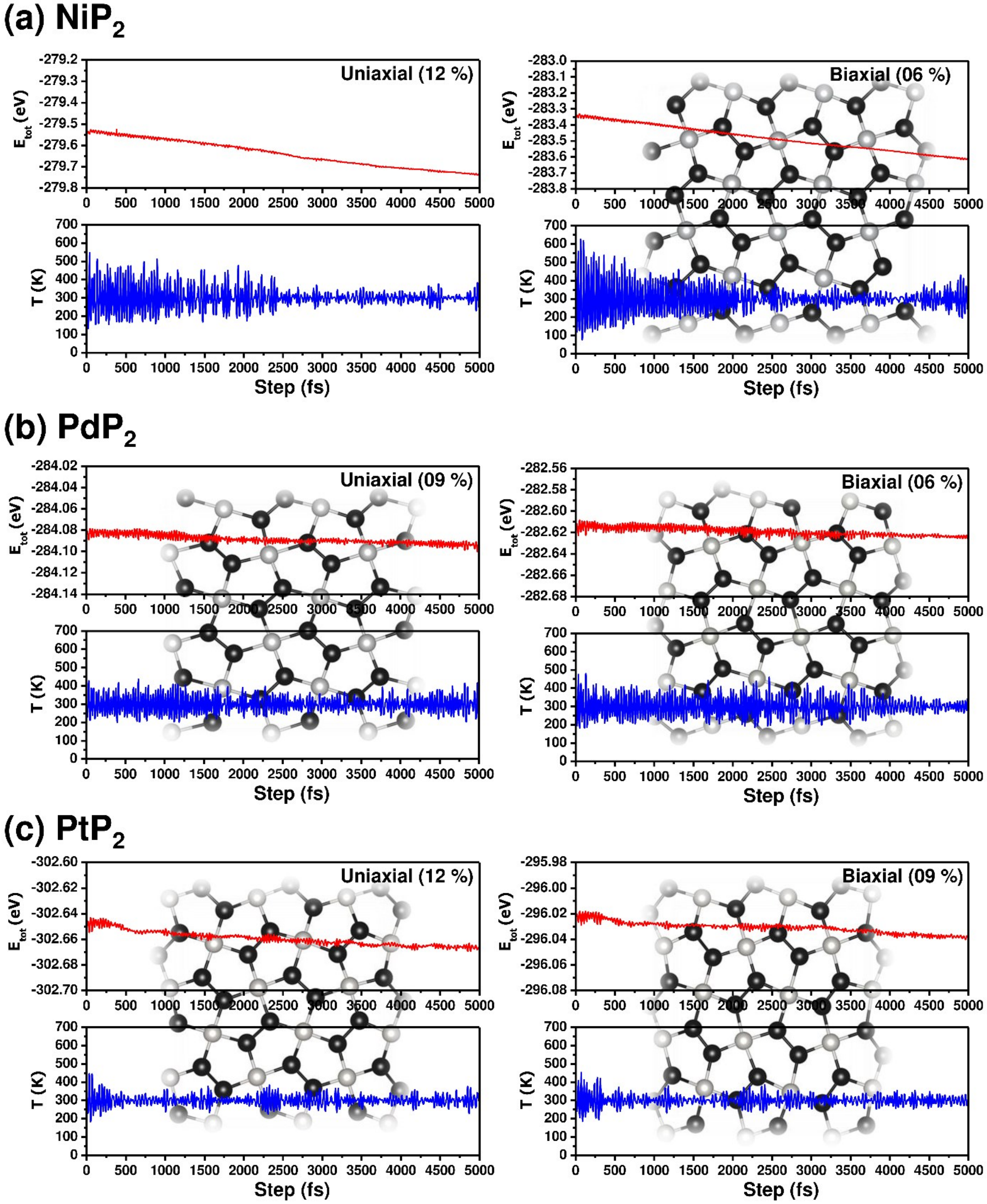

Fig. S7: The AIMD profile of fluctuation in total potential energy (E_{tot}) and temperature for both the uni- and biaxial optimum strains of the Penta-MP_2 compounds at 300 K temperature. The snapshots represented are at the end of 5 ps AIMD simulations at 300 K.

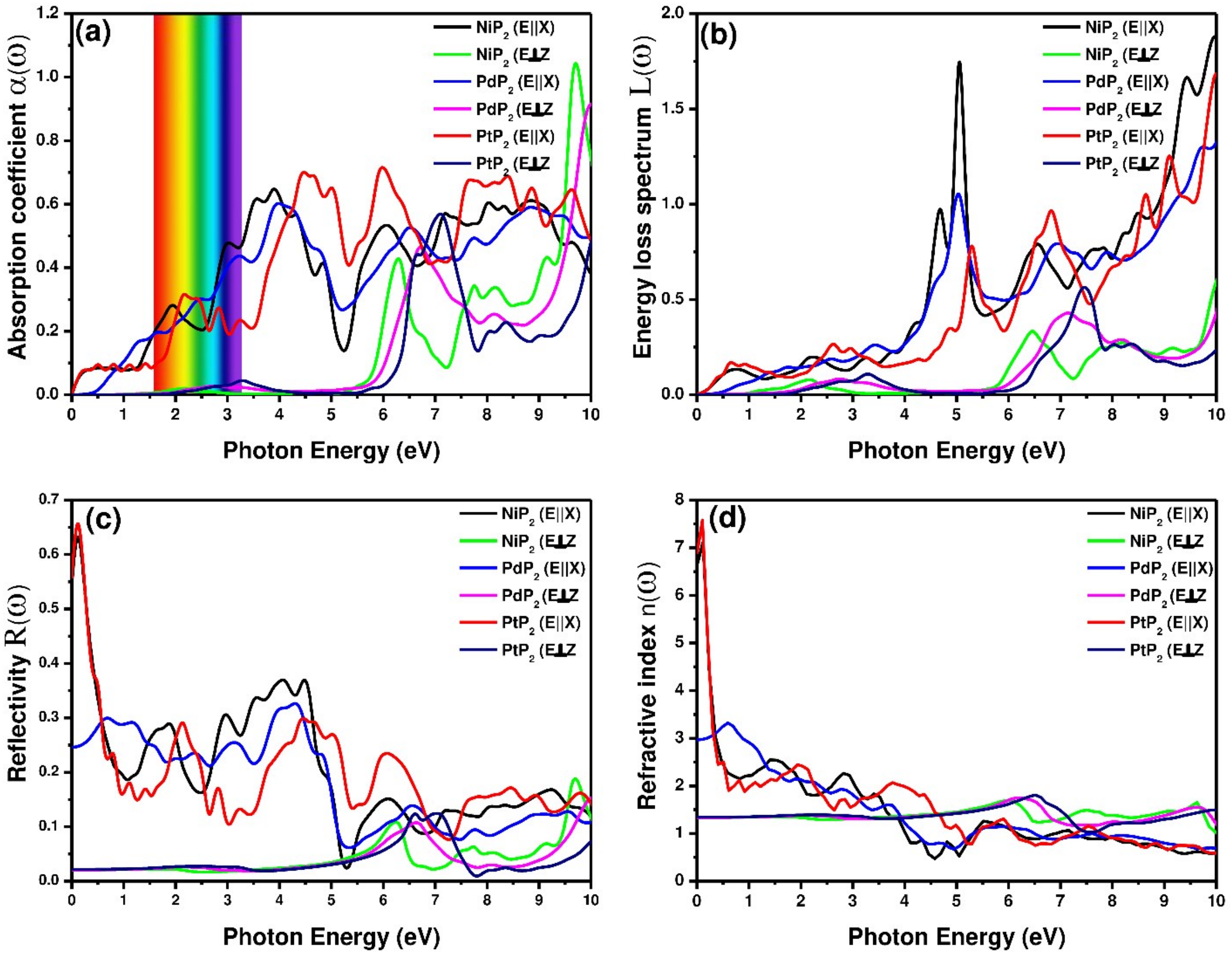

Fig. S8: The profiles of optical properties of Penta-MP2 (M=Ni, Pd, Pt) monolayers without strain: (a) Absorption coefficient (10^5), (b) Energy loss spectrum, (c) Reflectivity and (d) Refractive index.
